# Deficiency in STAT1 Signaling Predisposes Gut Inflammation and Prompts Colorectal Cancer Development

**DOI:** 10.3390/cancers10090341

**Published:** 2018-09-19

**Authors:** Sonia Leon-Cabrera, Armando Vázquez-Sandoval, Emmanuel Molina-Guzman, Yael Delgado-Ramirez, Norma L. Delgado-Buenrostro, Blanca E. Callejas, Yolanda I. Chirino, Carlos Pérez-Plasencia, Miriam Rodríguez-Sosa, Jonadab E. Olguín, Citlaltepetl Salinas, Abhay R. Satoskar, Luis I. Terrazas

**Affiliations:** 1 Unidad de Biomedicina. Facultad de Estudios Superiores-Iztacala, Universidad Nacional Autónoma de México (UNAM), Av. De los Barrios 1, Los Reyes Iztacala, Tlalnepantla, Edo. De México 54090, Mexico; soleon81@gmail.com (S.L.-C.); armiin.vazsan@gmail.com (A.V.-S.); dayaknegro@gmail.com (E.M.-G.); yael_dery@hotmail.com (Y.D.-R.); nldb1@hotmail.com (N.L.D.-B.); aletse_bianca@hotmail.com (B.E.C.); irasemachirino@gmail.com (Y.I.C.); carlos.pplas@gmail.com (C.P.-P.); rodriguezm@unam.mx (M.R.-S.); efra004@gmail.com (J.E.O.); 2 Carrera de Médico Cirujano, Facultad de Estudios Superiores Iztacala, UNAM, Av. De los Barrios 1, Los Reyes Iztacala, Tlalnepantla, Edo. De México 54090, Mexico; cisala69@hotmail.com; 3 Unidad de Investigación Biomédica en Cáncer, UNAM-Instituto Nacional de Cancerología, Ciudad de México 14080, Mexico; 4 Laboratorio Nacional en Salud, Facultad de Estudios Superiores-Iztacala, UNAM, Edo. De México 54090, Mexico; 5 Department of Pathology, The Ohio State University, Columbus, OH 43210, USA; Abhay.Satoskar@osumc.edu

**Keywords:** colorectal cancer, STAT1, inflammation

## Abstract

Signal transducer and activator of transcription 1 (STAT1) is part of the Janus kinase (JAK/STAT) signaling pathway that controls critical events in intestinal immune function related to innate and adaptive immunity. Recent studies have implicated STAT1 in tumor–stroma interactions, and its expression and activity are perturbed during colon cancer. However, the role of STAT1 during the initiation of inflammation-associated cancer is not clearly understood. To determine the role of STAT1 in colitis-associated colorectal cancer (CAC), we analyzed the tumor development and kinetics of cell recruitment in wild-type WT or STAT1^−/−^ mice treated with azoxymethane (AOM) and dextran sodium sulfate (DSS). Following CAC induction, STAT1^−/−^ mice displayed an accelerated appearance of inflammation and tumor formation, and increased damage and scores on the disease activity index (DAI) as early as 20 days after AOM-DSS exposure compared to their WT counterparts. STAT1^−/−^ mice showed elevated colonic epithelial cell proliferation in early stages of injury-induced tumor formation and decreased apoptosis in advanced tumors with over-expression of the anti-apoptotic protein Bcl2 at the colon. STAT1^−/−^ mice showed increased accumulation of Ly6G^+^Ly6C^−^CD11b^+^ cells in the spleen at 20 days of CAC development with concomitant increases in the production of IL-17A, IL-17F, and IL-22 cytokines compared to WT mice. Our findings suggest that STAT1 plays a role as a tumor suppressor molecule in inflammation-associated carcinogenesis, particularly during the very early stages of CAC initiation, modulating immune responses as well as controlling mechanisms such as apoptosis and cell proliferation.

## 1. Introduction

Colorectal cancer is one of the most frequent neoplasms and is the second most common cause of death by cancer in Western countries [1]. Colitis-associated colon cancer (CAC), which develops under chronic inflammatory conditions in the intestinal tissue, is different from sporadic cancer and is one of the most frequent causes of morbidity and mortality in inflammatory bowel disease (IBD) patients [2,3]. Ulcerative colitis (UC) and Crohn’s disease (CD) are considered the main components of human IBD. Patients with UC have an increased risk of developing CAC, and the extent and duration of the disease augment its threat. After 8–10 years of diagnosis of IBD, the risk of developing CAC augments to 2–40% compared to that of the general population, depending on the severity and location of IBD [4,5]. In addition, CAC is considered to have greater malignant potential than sporadic colorectal cancer and patients show poor survival rates in the advanced stages [6]. However, the immunological mediators underlying the development and progression of colorectal cancer preceded by chronic inflammation are still unclear. 

During the transformation process, inflammatory cytokines are constantly present in the intestinal tissue, and many of these cytokines generate cellular effects and functions through the Janus kinases (JAK)/signal transducer and activator of transcription (STAT) signaling pathway. The JAK/STAT pathway controls important events in intestinal immune function as well as in inflammatory responses [7], and its role in colorectal cancer development is just starting to be understood [8]. The binding of type I or type II interferons (IFNs) induces oligomerization of their receptor and activation of Janus kinases (JAKs), Jak1 and Jak2. The activation of these proteins favors the phosphorylation of the intracellular domain of the receptor which function as a docking site for signal transducer and activator of transcription 1 (STAT1) proteins. STAT1 forms homo- or hetero-dimers and translocates to the nucleus to initiate the transcription of a repertoire of target genes [9]. Studies in mice and data from human patients have suggested that STAT1 has tumor suppressor properties; however, there is growing evidence that it can also act as a tumor promoter [10,11]. A decrease or loss of STAT1 signaling activity has been reported in many types of cancer, such as colon cancer [8], breast cancer [12], leukemia, and melanoma [13] and a correlation between the high expression of STAT1 and good prognosis has been observed in colorectal [14,15], pancreatic [16], and esophageal cancers [17]. 

However, high levels of STAT1 expression and activation in mucosal samples have been observed in patients with UC and CD [18] which may account for accelerated colon cancer development. In contrast, higher levels of total STAT1 but not phospho-STAT1 were observed in patients with CD compared to UC and controls [19]. Dextran sodium sulfate (DSS)-induced colitis was attenuated in STAT1^−/−^ mice who had significantly decreased tissue damage and hyaluronan (a molecule involved in leukocyte attachment) deposition at the intestines [20]. Additionally, a decrease in Th1-type cytokine production and a reduction in chemically-induced colitis was observed in mice when pharmacological inhibition of STAT1 was performed [21]. Suppressor of cytokine signaling 1 (SOCS1) acts as an important negative regulator for IFN-γ signaling by binding to JAKs and inhibiting their function. The restriction of IFN-γ-STAT1 signaling by SOCS1 seems to be necessary for the maintenance of Foxp3 expression and regulatory T cell integrity and function [22], an essential population that protects the host from excessive immune responses. Colons from SOCS1 deficient mice exhibited hyperactivation of STAT1, accompanied with an increased in carcinogenesis-related enzymes, cyclooxygenase-2, and inducible nitric oxide and showed spontaneous development of colorectal carcinomas [23]. Nevertheless, some evidence suggests that STAT1 seems to have a protective function in CAC development; for example, normal intestinal fibroblasts inhibited the proliferation of colon cancer cells via stimulation of STAT1 signaling in contrast to fibroblasts isolated from CD, UC, or colon cancer patients [24]. STAT1 has been shown to mediate the anti-proliferative effect of IFN-γ on tumor cells [25], and STAT1 is an important mediator for antiangiogenic signals, and inhibits tumor growth and metastasis of RAD-105 cells in vivo [26]. A recent report showed that pro-oncogenic activity of SOCS1 is related to the down-modulation of STAT1 expression [27]. Furthermore, active KRAS mutations in colon cancer cells were shown to inhibit the expression of STAT1, allowing them to escape tumor surveillance mechanisms due to reduced responsiveness to IFN-γ [28]. However, the role of STAT1 during inflammation-associated colon cancer has not been well stablished.

In this study, we demonstrate, for the first time, that STAT1 signaling has an important role in vivo during the development of CAC. Early STAT1 deficiency promotes rapid and extensive intestinal damage, increased proliferation in the early stages of injury-induced tumor formation, and reduced apoptosis in advanced tumors. A lack of STAT1 renders mice highly susceptible to CAC which correlates with dysregulation in the recruitment of monocytic and granulocytic cells. Thus, STAT1 signaling may represent a potential target for therapeutic intervention during the initial stages of colorectal cancer. 

## 2. Results 

### 2.1. STAT1 Deficiency Leads to Rapidly Increase Development of CAC

**Figure 1:**
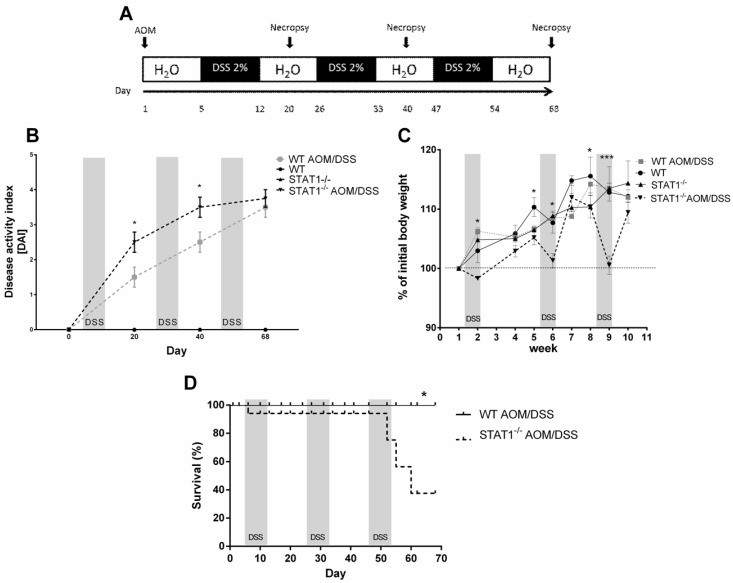
Changes during the course of colorectal cancer (CAC) in WT and signal transducer and activator of transcription (STAT1) deficient mice (STAT1^−/−^). (**A**) Schematic time schedule of azoxymethane (AOM) and dextran sodium sulfate (DSS) administration. After the initial AOM injection (12.5 mg/kg), DSS was given in drinking water for seven days followed by 14 days of regular drinking water. Mice were sacrificed on Days 20, 40 (early tumor development), and 68 (late tumor development) post AOM injection. (**B**) The disease activity index score (DAI) was assessed on the indicated days for each animal and averaged per day for each group (mean ± SEM, n = 5 mice/group). (**C**) Weight changes in healthy mice and AOM/DSS-treated mice relative to initial body weights during the course of the experiment. The graph shows data from four independent experiments (mean ± SEM, n = 5 mice/group). One-way ANOVA and Tukey´s multiple comparisons tests were completed. * represents *p* ≤ 0.05, *** represents *p* ≤ 0.001. There was a significant difference between WT AOM/DSS versus STAT1^−/−^ AOM/DSS at the indicated time. (**D**) Survival curve comparing WT AOM/DSS (black solid line, n = 5) versus STAT1^−/−^ AOM/DSS WT (dashed line, n = 8). There was a significant difference in survival between WT AOM/DSS and STAT1^−/−^AOM/DSS (log rank test, * *p* = 0.0074). Data were pooled from two independent experiments.

**Figure 2:**
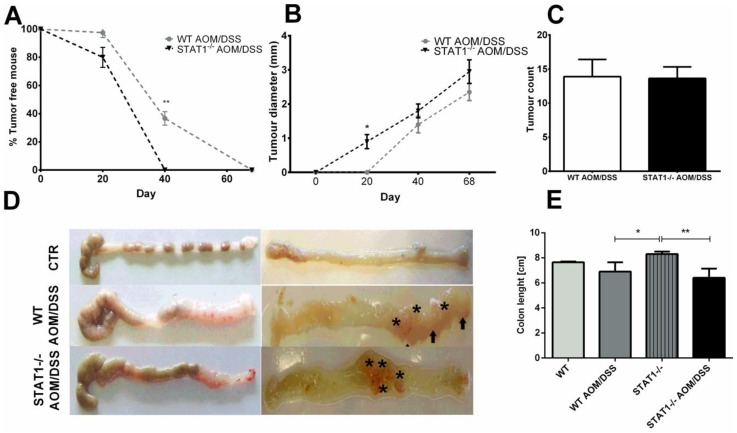
STAT1 deficiency leads to rapid development of CAC. Colons were removed and the number of macroscopic tumors were determined at the indicated time intervals. (**A**) Time course of the percentage of tumor-free mice during the AOM/DSS treatment in WT and STAT1^−/−^ mice. (**B**) Tumor diameter (mm) of colorectal tumors formed in WT AOM/DSS and STAT1^−/−^ AOM/DSS animals at the indicated times. (**C**) Number of colorectal tumors per mouse 68 days after AOM administration. (**D**) Representative photographs of the colon 68 days after the AOM injection (asterisks and arrows point to the tumors). (**E**) AOM/DSS treated mice had significantly smaller colons than control mice. Total data (**A**,**B**,**C**,**E**) and representative data (**D**) of two independent experiments with at least three mice per group. Data are expressed as means ± SDs. Statistically significant differences between two groups were judged by the Student’s *t*-test. * *p* ≤ 0.05, ** *p* ≤ 0.01.

To determine whether STAT1 participates in the development of CAC, we subjected WT and STAT1 deficient animals (STAT1^−/−^) to an azoxymethane (AOM)/DSS regiment, a well-established previously reported protocol [29]. Single administration of AOM and three cycles of DSS exposure induced colon tumorigenesis in mice with chronic colitis. We evaluated the course of the disease for 20-, 40-, and 68-day periods in STAT1^−/−^ and WT mice (Figure 1A) as an approximation of different stages of tumor progression. First, we monitored weight loss, stool consistency, and bleeding as the DAI score (Figure 1B). STAT1^−/−^ AOM/DSS mice featured an early and dramatic enhancement in diarrhea and rectal bleeding, and a reduction in weight at the end of every DSS cycle compared to similarly treated WT animals (Figure 1B,C). Additionally, AOM/DSS administration in STAT1^−/−^ mice resulted in significantly reduced early survival during the first and third DSS cycles (Figure 1D). At this early stage (Day 20), at least 20% of STAT1^−/−^ AOM/DSS mice displayed small colon tumors (Figure 2A,B), whereas WT AOM/DSS animals did not show any tumors. By Day 40, 100% of STAT1^−/−^ AOM/DSS mice had developed tumors, while, in the WT AOM/DSS group, only 70% of mice had tumors (Figure 2A). Interestingly, at necropsy on Day 68, 100% of STAT1^−/−^ and WT AOM/DSS treated animals presented reddish polypoid tumors at the medial and distal zones of the colon with no differences in size and number between groups (Figure 2A–E). 

### 2.2. STAT1^−/−^ AOM/DSS Animals Have More Histological Damage Compared to WT AOM/DSS Mice

**Figure 3:**
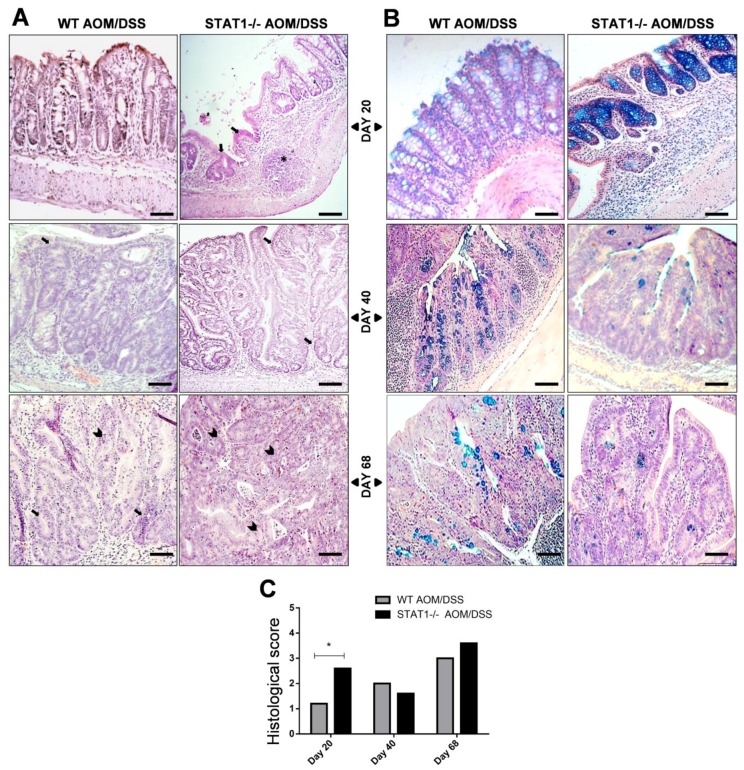
STAT1 deficient mice have more advanced dysplasia and goblet cell decreases compared to WT colons. The colons were removed, fixed, and stained with H&E (**A**) or Alcian blue (**B**) at the indicated time intervals. (**C**) The histological score shown in each group of mice was determined as mentioned in M&M and is expressed as mean ± SD. Scale bars: 50 µm. * indicate hyperplastic lymphoid node, arrow indicate numerous hyperplastic gland polyps and arrowhead atypical epithelial cells with dysplastic nucleus and mitotic figures. A remarkable decrease of goblet cells was observed at Day 40 in STAT1^−/−^ AOM/DSS. Demonstrative photographs and data are representative of two independent experiments with at least three mice per group per day of the analysis. Statistically significant differences between two groups were judged by Student’s *t*-tests. * *p* ≤ 0.05.

To investigate the impact of STAT1 deficiency on tumor progression, a histological analysis of the colons was performed. Microscopic examination showed that STAT1^−/−^ AOM/DSS mice developed significant severe inflammation with massive infiltration of leucocytes into the mucosa, and extensive ulceration and erosion, particularly in the middle to distal colon at Day 20 after AOM administration compared with WT AOM/DSS animals (Figure 3A,C). In conjunction with increased histologic damage at this time, STAT1^−/−^ AOM/DSS mice showed increased DAI and a 10% reduction in survival (Figure 1B,D), demonstrating that the loss of STAT1 reduces the resistance to AOM/DSS CAC. At Day 40, inflammatory cell infiltration persisted in both treated groups, accompanied by dystrophic goblet cells, decreased mucin production, and loss of crypts and surface epithelium (Figure 3A–C). At Day 68, histological analyses revealed dysplastic glands with hyperchromatic nuclei and dystrophic goblet cells that were compatible with well-differentiated adenocarcinomas in all mice that received the AOM/DSS regimen (Figure 3A–C). However, the severity and extent of tissue damage were more pronounced in STAT1^−/−^ AOM/DSS mice, where 90% of analyzed tissue was affected. In contrast, in WT AOM/DSS mice, high-grade-dysplasia areas were alternated with normal areas. No evidence of damage was noted in WT or STAT1^−/−^ control mice.

### 2.3. The Absence of STAT1 Does Not Alter the Early Expression of Markers for Tumorigenesis

**Figure 4:**
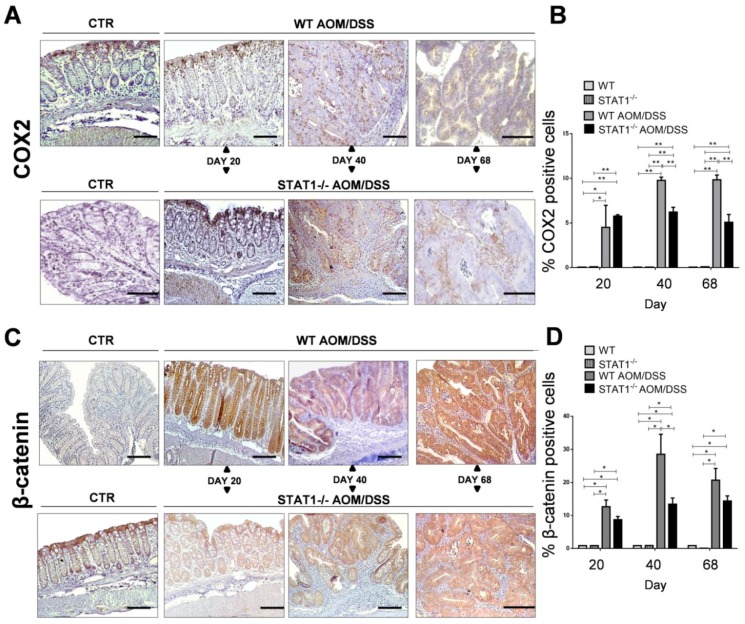
Immunohistochemical detection of COX-2 and β-catenin in colon tissue. Distal/affected colons were obtained from WT and STAT1^−/−^ AOM/DSS-treated or control mice at the indicated time intervals. (**A**) Immunostaining for cyclooxygenase-2 enzyme (COX-2) in the colons of control and treated groups. (**B**) Average percentages of COX-2^+^ cells in distal colon samples are indicated in the bar graph. (**C**) Immunostaining for β-catenin. (**D**) Average percentages of β-catenin+ cells in the colon (scale bars, 50 µm). Quantification of COX2^+^ and β-catenin^+^ cells was performed using ImageJ software v.1.48 by counting cells in 10 high-powered fields in at least three slides per animal. Data are expressed as mean ± SEM and are representative of two independent experiments with at least three mice per group per day of the analysis. * *p* < 0.05, ** *p* < 0.01.

β-Catenin and cyclooxygenase-2 enzyme (COX-2) play essential roles in colon carcinogenesis. Increased expression of both could be used as a marker of tumor progression and poor prognosis [29]. A significant increase in COX-2 expression was observed in WT AOM/DSS and STAT1^−/−^ AOM/DSS mice compared with control mice in immunohistochemical staining (Figure 4A,B). However, the percentage of COX-2^+^ cells in the colon tissue was significantly lower in STAT1^−/−^ AOM/DSS mice compared with WT AOM/DSS animals at 40 and 68 days after AOM administration (Figure 4A,B). As expected, strongly positive cytoplasmic or nuclear β-catenin staining was observed in transformed tissue in WT and STAT1^−/−^ AOM/DSS treated mice compared with the control groups (Figure 4C,D). At 20 and 68 days after AOM administration, the percentage of β-catenin+ cells was similar in WT AOM/DSS and STAT1^−/−^ AOM/DSS animals. Conversely, at Day 40, we observed a significant decrease in the percentage of β-catenin+ cells in STAT1^−/−^ AOM/DSS animals compared with WT AOM/DSS mice. These results suggest that STAT1 deficiency is related to colorectal cancer initiation and/or promotion.

### 2.4. STAT1 Deficiency Increases Cell Proliferation and Reduces Apoptosis during Early CAC Development

**Figure 5:**
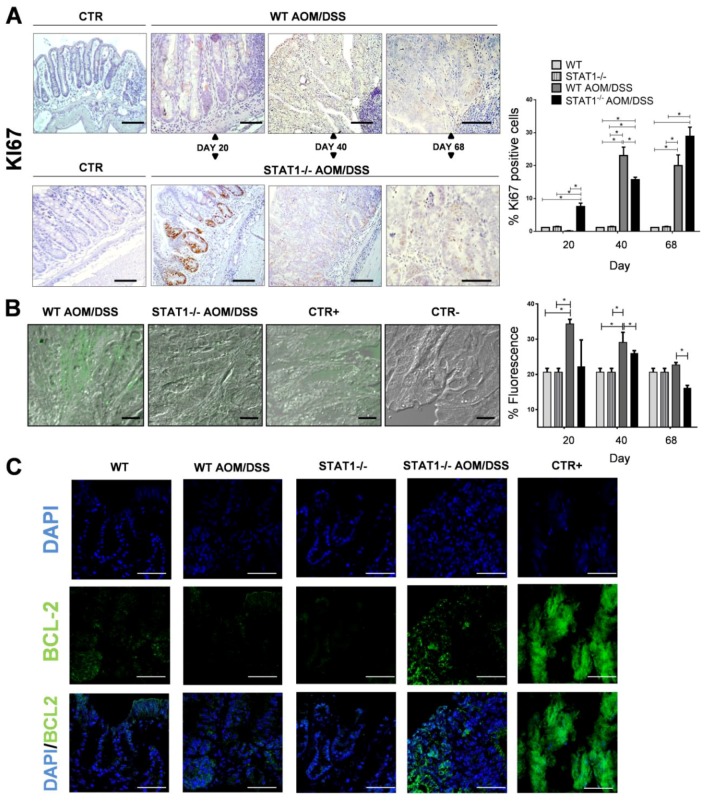
Enhanced colon epithelial cell proliferation and reduced apoptosis in STAT1^−/−^ mice during CAC development. Distal/affected colons were obtained from WT and STAT1^−/−^AOM/DSS-treated or control mice at the indicated time intervals. (**A**) Representative Ki67 immunohistochemistry (left) in WT and STAT1^−/−^ mice given AOM/DSS or control groups. Scale bars: 50 µm. Ki67^+^ cells were quantified using ImageJ software v.1.48 by counting cells in 10 high-powered fields in at least three slides per animal (right). (**B**) Apoptotic cells in distal colons from WT and STAT1^−/−^ control and AOM/DSS treated mice were monitored by an in situ cell death assay in which cleavage of genomic DNA during apoptosis yields double strand as well as single strand breaks (nicks), and then were identified by labeling free 3′-OH terminal with fluorescein. Representative images at 20 days after AOM injections are shown (left). Scale bars: 20 µm. The positive control was treated with DNase enzyme, while in the negative control; the cells were incubated with label solution only. Data are presented as the percentage of mean fluorescence, which was expressed as arbitrary units (right). (**C**) Representative images of immunofluorescence staining of Bcl-2 in the distal/affected colon tissue of the indicated experimental groups. The thymus was used as a positive control. Scale bars: 20 µm. Data are expressed as mean ± SEM and are representative of two independent experiments with at least 3 mice per group per day of the analysis. * *p* < 0.05, ** *p* < 0.01.

One potential mechanism contributing to accelerated tumorigenesis in STAT1^−/−^ mice is the increased proliferation and reduced apoptosis in the mucosa and tumors of these mice. To test this hypothesis, we analyzed the expression of Ki67, a marker of cell proliferation, in intestinal epithelial cells at Days 20, 40, and 68 of the AOM/DSS regimen in WT and STAT1^−/−^ mice. The immunohistochemistry of Ki67 showed a significant increase in the number of proliferating cells stained with anti-Ki67 antibody in colon crypts of STAT1^−/−^ AOM/DSS mice compared with WT AOM/DSS animals at Day 20 of AOM injection (Figure 5A). There was no substantial difference in the number of Ki67+ cells between control groups. In advanced stages of tumor development, at Days 40 and 68, the number of Ki67^+^ cells also increased in both WT and STAT1^−/−^ AOM/DSS treated mice. However, at Day 40, STAT1^−/−^ AOM/DSS mice presented less proliferation than WT AOM/DSS animals. At Day 68, no substantial differences in the number of Ki67^+^ cells were observed between groups (Figure 5A). These data demonstrated that the proliferation of the intestinal epithelium is augmented in STAT1^−/−^ mice that receive AOM/DSS treatment during the early stages of CAC development. 

Next, to analyze the state of epithelial apoptosis between WT and STAT1^−/−^ mice during AOM/DSS administration, we performed the in situ terminal deoxynucleotidyl transferase-mediated dUTP nick-end labeling (TUNEL) assay on colon sections on Days 20, 40, and 68. WT AOM/DSS animals showed an increased percentage of TUNEL-positive cells in the early stages of CAC development (Days 20 and 40) compared with control mice (*p* < 0.05) (Figure 5B). TUNEL-positive cells were seen in the surface epithelium of the colon in both WT AOM/DSS and STAT1^−/−^ AOM/DSS animals, and there was no difference in the number of apoptotic cells at Day 20. However, TUNEL-positive cells were significantly decreased in STAT1^−/−^ AOM/DSS mice compared with WT AOM/DSS mice at Day 40 and 68 (Figure 5B). No differences were observed between control groups. 

To investigate the possible involvement of STAT1 in apoptosis control during CAC, we evaluated the protein expression of Bcl-2 at the distal/affected part of the colon. The Bcl-2 family includes proteins that play central roles in cell death regulation, and they are altered in cancer [30]. Recently, it was reported that STAT1 is able to modulate apoptosis regulators like Bcl-2 [31]. To detect Bcl-2 expression at the tumor site, we performed immunofluorescence on colon slides. As expected, immunofluorescence of Bcl-2 showed greater amounts of this protein in STAT1^−/−^ AOM/DSS colons than in WT AOM/DSS and control colons (Figure 5C). These results suggest that STAT1 is related to the balance of proliferation and apoptosis in the colon during CAC development. 

### 2.5. STAT1 Deficiency Alters the Expression of Proinflammatory Mediators in the Colon

**Figure 6:**
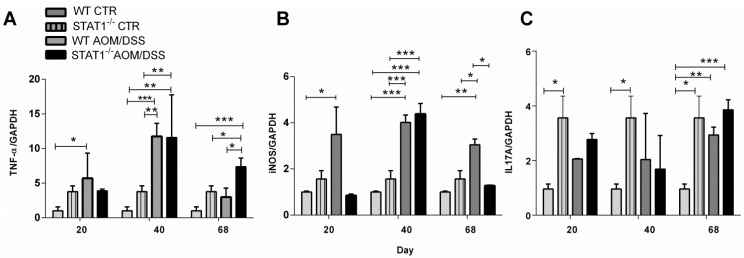
STAT1 can modulate the inflammatory responses involved in tumor development. RT-PCR analyses of (**A**) TNF-α, (**B**) IL-17A, and (**C**) iNOS were performed on total RNA extracted from distal colons at indicated times and were normalized to GAPDH mRNA levels. Data are expressed as mean ± SEM and are representative of two independent experiments with at least three mice per group per day of the analysis. * *p* < 0.05, ** *p* < 0.01, *** *p* < 0.001.

To determine if some critical cytokines and inflammatory factors such as TNF-α, IL-17A, and iNOS, which are involved in inflammation and tumor progression, were altered during CAC in STAT1 deficiency, we analyzed their gene expression in distal colon samples by RT-PCR in both control and CAC-induced mice. As shown in Figure 6A,B we observed a significant increase in TNF-α and iNOS expression in WT AOM/DSS colons compared with control colons during the development of CAC. This increase was observed from Day 20 until Day 68. In contrast, in STAT1 AOM/DSS mice, significant increases in the transcription levels of TNF-α and iNOS were observed later, at Day 40, suggesting that at early times, other inflammatory mechanisms may take place in the absence of STAT1 (Figure 6A,B). We also examined IL-17A transcripts (Figure 6C). Our results indicated that STAT1^−/−^ mice expressed higher IL-17A transcripts than WT mice (Figure 6C). Additionally, IL-17A expression was slightly higher in STAT1^−/−^ AOM/DSS colons compared with WT AOM/DSS colons at 20 and 68 days, although this difference was not statistically significant.

### 2.6. The Recruitment of Inflammatory Monocytes and Granulocytes during CAC Is STAT1 Dependent

**Figure 7:**
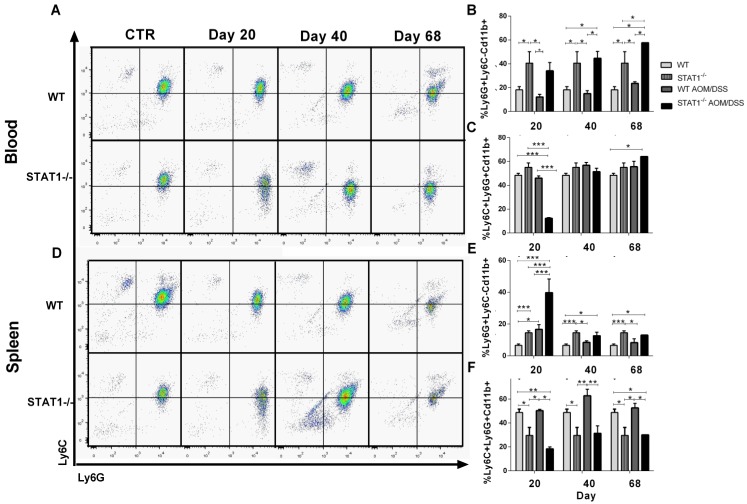
Increase of Ly6G^+^Ly6C^−^Cd11b^+^ cells in STAT1^−/−^ mice during CAC development. Circulating and spleen cells were obtained from WT or STAT1^−/−^AOM/DSS-treated or control mice at the indicated time intervals and were analyzed for expression of Ly6C and Ly6G on living CD11b^+^ population cells by flow cytometry. (**A**) Representative dot plots in total blood. (**B**) Frequencies of Ly6G^+^Ly6C^−^CD11b^+^ cells and (**C**) Ly6C^+^Ly6G^+^CD11b^+^ cells in the blood of WT or STAT1^−/−^ AOM/DSS-treated or control mice, gated as in (**A**). (**D**) Representative dot plots in splenocytes. Frequencies of (**E**) Ly6G^+^Ly6C^−^CD11b^+^ cells and (**F**) Ly6C^+^Ly6G^+^CD11b^+^ cells in the splenocytes of WT or STAT1^−/−^ AOM/DSS-treated or control mice, gated as in (**D**). Data are expressed as mean ± SEM and are representative of two independent experiments with at least three mice per group per day of the analysis. * *p* < 0.05, ** *p* < 0.01, *** *p* < 0.001.

Monocytes and granulocytes can be recruited during tumor development and can alter tumor specific immune defense mechanisms [32]. To investigate whether the deletion of STAT1 results in compensatory accumulation of monocytes and granulocytes, as well as in promoting inflammation and tumor growth in STAT1^−/−^ AOM/DSS mice, we evaluated the expression of CD11b, Ly6C, and Ly6G in the spleens and peripheral blood. As shown in Figure 7, two subsets of CD11b+ cells were distinguished in the spleens and blood of mice—a major one, marked as Ly6C+Ly6G+CD11b+, and a minor one, marked as Ly6G^+^Ly6C^−^CD11b^+^. The abundance of these populations was dependent on STAT1. Ly6G^+^Ly6C^−^CD11b^+^ cells were detected at a high frequency in the blood of control and AOM/DSS treated animals with STAT1 deficiency (Figure 7A,B) and increased as CAC progressed. In contrast, this population was significantly less abundant in WT AOM/DSS animals. In spleen cells, STAT1^−/−^ AOM/DSS mice presented enhanced accumulation of Ly6G^+^Ly6C^−^CD11b^+^ cells compared with WT AOM/DSS and control mice at 20 days of AOM injection. We also observed an increased frequency of Ly6G^+^Ly6C^−^CD11b^+^ in the spleens of control STAT1^−/−^ mice compared with WT counterparts (Figure 7D,E). In addition, circulating Ly6C^+^Ly6G^+^CD11b^+^ and spleen cells were significantly less abundant in STAT1^−/−^ AOM/DSS mice compared with WT AOM/DSS at 20 days of CAC progression (Figure 7A–C,F). The spleens of WT AOM/DSS mice showed enhanced accumulation of Ly6C^+^Ly6G^+^CD11b^+^ cells at Days 40 and 68 compared with STAT1 AOM/DSS mice (Figure 7D,F). Thus, the absence of STAT1 resulted in increased accumulation of granulocytic myeloid cells (GMCs), particularly at Day 20. 

### 2.7. IL-17A, IL-17F and IL-22 Cytokine Production Is Increased during STAT1 Deficiency

**Figure 8:**
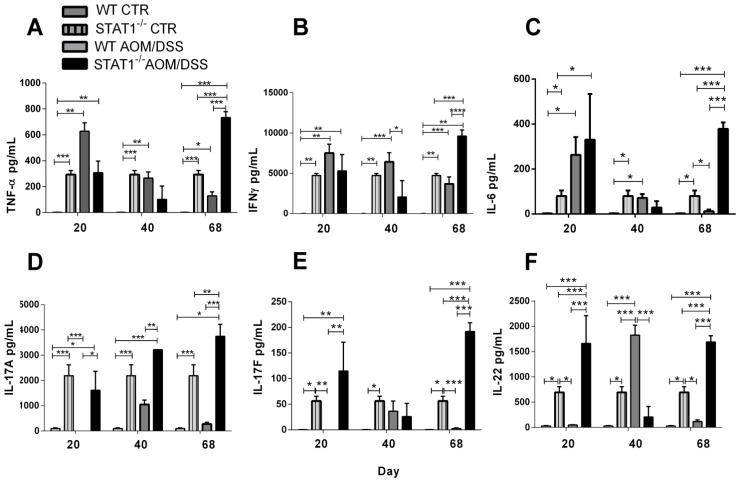
The deficiency of STAT1 alters cytokine production during CAC development. (**A**) TNF-α, (**B**) IFN-γ; (**C**) IL-6; (**D**) IL-17A; (**E**) IL-17F; and (**F**) IL-22 cytokine production from splenocytes of control or CAC-induced WT and STAT1^−/−^ mice stimulated for 48 h with anti-CD3 antibody. Data are expressed as mean ± SEM and are representative of two independent experiments with at least three mice per group per day of the analysis. * *p* < 0.05, ** *p* < 0.01, *** *p* < 0.001.

The increase in Ly6G^+^CD11b^+^ cells is probably the result of cytokines promoting their development and accumulation. Recent reports have shown that the expansion of peripheral GMCs is correlated with higher stages and histological grades of colon cancer, suggesting that they play a role in colon cancer progression [33]. To determine whether cytokine production was involved in the observed accumulation of Ly6G^+^Ly6C^−^CD11b^+^ cells in the spleens, as well as in increased development of CAC in STAT1^−/−^ mice, we exanimated TNF-α, IFN-γ, IL-6, IL-17A, IL-17F, and IL-22 cytokine production in stimulated splenocytes in both control and CAC-induced mice (Figure 8). Our results indicated that TNF-α, IFN-γ, and IL-6 levels were significantly increased at Day 20 in WT AOM/DSS mice compared with WT mice. As CAC progressed, the production of these cytokines decreased (Figure 8A–C). On the contrary, spleen cells from STAT1^−/−^ AOM/DSS mice showed lower levels of TNF-α and IFN-γ at Days 20 and 40, but there were increased levels of these cytokines at Day 68 (Figure 8A–C). Interestingly, the analysis of IL-17A, IL-17F, and IL-22 cytokine production indicated that STAT1 AOM/DSS stimulated splenocytes produced higher levels of these cytokines than WT AOM/DSS splenocytes (Figure 8D–F) as CAC progressed. Interestingly, STAT1^−/−^ mice were shown to intrinsically produce more Th-17 cytokines in T cells stimulated with anti-CD3 antibody in vitro which was enhanced by AOM/DSS administration.

## 3. Discussion

Emerging data show the importance of the JAK/STAT signaling pathway in the control of important events in intestinal homeostasis, like cell differentiation, the secretion of cytokines, and proliferation and apoptosis during IBD and colorectal cancer [34,35,36]. The tumor suppressor function of STAT1 in colorectal cancer development and progression has been established [10]. Some studies reported longer survival rates in CRC patients with high nuclear STAT1 and low nuclear STAT3 levels [37], and STAT1 was identified as belonging to a group of tightly co-regulated immune related genes that influence the tumor immunophenotype and are linked with disease-free survival in a cohort of colon cancer patients [10]. However, the mechanisms through which STAT1 might exert that function remain controversial. We investigated the relevance of STAT1 to intestinal tumorigenesis under inflammatory conditions and evaluated the role of immune cells which, in conjunction with STAT1, are important for the prevention of CAC. 

In our experimental model, in the absence of STAT1, tumor appearance was faster, suggesting a favorable microenvironment for tumor development. Persistent activation of STAT3 is frequent in maligned cells, and STAT3 signaling induced the expression of genes that are important for cancer inflammation [38]. STAT1 signaling can modulate STAT3 function by different mechanisms. A previous study showed that CRC cell lines with a low STAT1/high STAT3 ratio presented faster tumor growth when they were xenografted into SCID mice. On the contrary, decreased STAT3 signaling caused an augment in STAT1 expression and reduced tumor growth [37]. In accordance with this finding, we observed increased cell proliferation at the initial steps of tumor development in STAT1^−/−^ CAC-induced colons, consistent with more signs of the disease, damage, and reduced survival. Considering that STAT1 can antagonize STAT3 function, the complete knockout nature of STAT1 mice could favor the persistent activation of STAT3, increasing colon tumorigenesis. Additionally, in advanced stages of the disease, STAT1^−/−^ tumors exhibited a significantly reduced percentage of apoptotic cells accompanied by an increased level of the anti-apoptotic protein Bcl-2. These data highlight that the absence of STAT1 results in a decreased ability to control the expression of pro-survival proteins, such as Bcl-2, during colorectal cancer. In fact, STAT1 can inhibit the transcription of anti-apoptotic members of the Bcl-2 family of proteins [39]. However, we did not observe any differences in the number of tumors at the end of the experiment, suggesting that other mechanisms were taking place.

Even though STAT1 deficient mice have more advanced dysplasia and a greater decrease in goblet cells compared to WT colons, the absence of STAT1 does not alter the early expression of markers of tumorigenesis—COX2 and β-catenin—with the same magnitude in WT animals. COX2 and other mediators are critical for maintaining a cancer promoting inflammatory milieu, and they are mostly produced by the stromal inflammatory cells. In the absence of STAT1, other oncogenic pathways could also participate. KRAS mutations in colorectal cancer cells are related to inactivation of STAT1 and decreased sensibility of tumor cells to the anti-tumorigenic effects of IFN-γ [27]. IFN-γ, thus, may inhibit tumor cell growth through a STAT1-dependent pathway [24]. 

In the present study, we found that WT CAC-induced animals with complete STAT1 signaling showed a significant increase in IFN-γ production by spleen cells and an augmentation in the transcription levels of iNOS and TNF-α in the colon during the early stages of injury and tumor formation, suggesting an effective Th1 response. In contrast, in STAT1^−/−^ CAC-induced mice, Th17 type cytokines increased, showing an imbalance in cytokine expression and production. At the intestines, IL-22 promotes survival, tissue healing, and intestinal epithelial homeostasis—functions which are critically dependent on STAT3. During experimental colitis, the ablation of IL-22/STAT3 signaling using tyrosine kinase 2 (TIK2) deficient mice exacerbates inflammatory bowel disease [40]. However, in our study, it was shown that IL-22 might have a detrimental role due to elevating the Th17 adaptive immune response. JAK1, JAK2 as well as TIK2 are activated in response to cytokines, and following the phosphorylation of STAT, proteins signal downstream target genes and establish autocrine and paracrine loops. Our results further support the idea that perturbations in STAT1 signaling not only affect apoptosis or survival functions, they seem to be important for maintaining cytokine balance and probably T cell function.

During CAC, in the absence of STAT1, we observed an important increase in Ly6G^+^Ly6C^−^CD11b^+^ circulating cells and their accumulation in the spleen at Day 20, suggesting that under these conditions, STAT1 signaling may play a critical role in the differentiation of myeloid cells. In our model, the increase in tissue damage and inflammation in STAT1^−/−^ CAC-induced mice, particularly in the initial steps of tumor development, indicates that another source of inflammation is induced when STAT1 is not present. Several studies have linked IL-17 production with colorectal cancer [41,42]. IL-17A deficient mice treated with the AOM/DSS regimen developed significantly less tumors and inflammatory mediators, such as IL-6, TNF-α, and IFN-γ, than WT mice [43]. In the present study, the absence of STAT1 significantly increased the production of Th17 cytokines (IL-17A, IL-17F, and IL-22) but not of TNF-α and IFN-γ, indicating that a lack of STAT1 signaling induces a significant change in the microenvironment that supports inflammation and tumor formation. 

Th17 responses are affected by STAT1-activating cytokines like IFN-γ and IL-27 [44]. Indeed, during systemic inflammation, STAT1 deficient T cells exhibited a hyper-Th17 phenotype relative to wild type controls [45]. This can explain the upregulated levels of IL-17A and IL-17F found in STAT1^−/−^ mice during the development of CAC. Moreover, IL-17A has been associated with susceptibility to CAC [43]. Thus, STAT1 may affect several other immunological pathways that may stop a Th17 inflammatory response in the local microenvironment. In line with this, the high levels of IL-17A produced by splenocytes from STAT1^−/−^ AOM/DSS mice may also favor the recruitment of granulocytes at the colon site as observed by accumulation in the spleen as well as at the circulatory level. In accordance with this finding, it has been well established that Th17 cytokines play a critical role in activating and recruiting neutrophils [46] and the enrollment of both monocytic and granulocytic cells with poor prognosis in different types of cancer, including CAC, has been reported [47,48]. 

Furthermore, we found a lack of recruitment of monocytic Ly6C^+^Ly6G^−^CD11b^+^ cells in STAT1^−/−^ mice, which have been proposed as the precursors of inflammatory M1 cells that may be involved in early inflammation and removal of recently transformed cells [49]. Thus, STAT1 appears to be necessary to recruit Ly6C^hi^ “inflammatory monocytes” early on in CAC development which may transform into effector M1 cells with cytotoxic activity in the tumor microenvironment. Instead, the absence of STAT1 appears to fuel the recruitment of Ly6G granulocytes which seems to reach the colon earlier than in WT mice. Granulocytic cells have been shown to be involved in the worsening of CAC development [50,51]. Therefore, STAT1 may play multiple roles in modulating CAC development—on one hand favoring the M1 response to block CAC development, and on the other hand, avoiding Th17 responses as well as neutrophil differentiation and recruitment. In addition, the high levels of IL-22 detected early on STAT1^−/−^ CAC-induced mice may also be associated with the rapid alterations in the colon and favor a more aggressive CAC. In accordance with our findings, it has been previously determined that excessive IL-22 production leads to tumor growth in colon cancer as well as apoptosis inhibition through STAT3 activation [52].

It has been reported that IFN-γ deficiency leads to the expansion of macrophages and granulocytes during infections and is, importantly, involved in hematopoiesis during bacterial infection [53,54]. Interestingly, IFN-γ^−/−^ mice infected with mycobacteria underwent splenic expansion of granulocytes and macrophages, and systemic levels of IL-6 and granulocyte colony-stimulating factor (G-CSF) were increased [53]. Recently, it has been suggested that, during the chronic stages of infection, IFNs, via STAT1, can have a predominantly suppressive role, inducing pathways involving IL-10 and programmed cell death ligand 1 (PDL1) [54]. In our study, DSS administration caused barrier dysfunction which led to enhanced susceptibility to bacterial invasion. STAT1 signaling seems to be important in limiting tissue damage in the context of chronic infections; however, there is no compelling evidence about STAT1’s involvement in this process during initial steps of CAC. The use of monocyte-specific knockout for STAT1 could be useful to help to resolve these questions. 

## 4. Materials and Methods

### 4.1. Mice 

Eight-week-old female BALB/c mice were purchased from ENVIGO (México) and maintained in a pathogen free environment at the Facultad de Estudios Superiores (FES) Iztacala, Universidad Nacional Autónoma de México animal facilities. STAT1^−/−^ female mice with a BALB/c genetic background were kindly donated by Dr. A.R. Satoskar. The animals were fed with Purina Diet 5015 (Purina, St. Louis, MO, USA) and water ad libitum. All experimental procedures were approved by the Ethical Committee of the Universidad Nacional Autónoma de México according to the University Animal Care and Use Committee: CE/FESI/102016/1096

### 4.2. Murine Colitis Associated Colorectal Cancer (CAC)

The CAC model was carried out as previously described [30]. Briefly, WT and STAT1^−^/^−^ mice received an intraperitoneal (i.p.) injection containing 12.5 mg/kg azoxymethane (AOM) (Sigma, St. Louis, MO, USA). Five days later, 2% dextran sodium sulfate (DSS, MW: 35,000–50,000, MP Biomedicals, Solon, OH, USA) was dissolved in the animals’ drinking water for 7 days. Afterwards, the mice received regular drinking water for 14 days. This experimental series was repeated twice. To examine the early and late transformative steps in CAC, the mice were killed on Days 20, 40 (early tumor development), and 68 (late tumor development) after AOM injection. Throughout the experiment, a Disease Activity Index (DAI) scores were given to evaluate the clinical progression of CAC. DAI scores were calculated as the sum of changes in weight loss compared to initial weight, stool consistency, and bleeding [55].

### 4.3. Histological Analysis 

Tissues collected from all experimental groups were fixed in 100% ethanol, processed, paraffin embedded and sectioned (5 μm). The tissue sections were stained with hematoxylin and eosin (H&E) solution (for pathological evaluation) and Alcian blue (for acidic polysaccharides). The histological evaluation was graded in a blinding manner, as follows: 0, no signs of inflammation; 1, low leukocyte infiltration, no more than 25%; 2, moderate leukocyte infiltration, more than 25% less than 50%; 3, high leukocyte infiltration, more than 50%; and 4, transmural infiltrations, no normal areas. Immunohistochemical staining for COX-2, β-catenin, Ki67, and immunofluorescence for Bcl-2, were performed using paraffin- embedded sections. Endogenous peroxidase was blocked using 3% H_2_O_2_ in methanol for 10 min. Antigen retrieval was performed using preheated citrate buffer (Biocare Medical, Diva Deloaker 10×) for 15 min. The slides were then washed with PBS-Tween and blocked using Albumin Bovine Serum 2% in PBS for 1 h. Then, the sections were incubated with the following antibodies (anti-COX2, 1:100, Genetex, Irvine, CA, USA; anti-β-catenin, 1:100, Genetex, Irvine, CA, USA and anti-Ki67, 1:100, Biolegend, San Diego, CA, USA). All sections were counterstained with hematoxylin. For immunofluorescence, tissue slides were incubated with primary antibodies (anti-Bcl2, 1:200, Biolegend) and were counterstained with DAPI (Abcam). The slides were mounted in EcoMount mounting medium (Biocare Medical, Concord, CA, USA) and were analyzed using an AxioVert.A1 Image capture optical microscope (Carl Zeiss Microscopy GmbH). Tissue microphotographs were captured using an AxioCam MRc and ZEN lite 2011 software v.1.0.1.0. Quantification of COX2^+^, β-catenin^+^, and Ki67^+^ cells was performed using ImageJ software v.1.48 by counting cells in 10 high-powered fields with at least three slides per animal. TUNEL staining was performed using the in situ Cell Death Detection Fluorescein kit (Roche, Indianapolis, IN, USA) in accordance with the manufacturer’s instructions. Samples were analyzed with a ZeissVert.A1 conventional epifluorescence microscope and a LEICA TCS SP8X confocal microscope (the analyzed area in each sample was 2.8 mm^2^, and 20 fields of 50 mm^2^ were evaluated). 

### 4.4. RNA Extraction and RT-PCR

Total RNA from colonic tissues was reverse transcribed and analyzed by semi quantitative PCR. The primer sequences and cycling conditions were as previously described [36].

### 4.5. Quantification of Cytokines 

Splenocytes from mice were plated in 96-well plates coated with anti CD3 (Biolegend, San Diego, CA, USA) antibodies (2 µg/mL) in complete RPMI medium in a humidified atmosphere containing 5% CO_2_ in air at 37 °C. After 24 h, the supernatants were harvested and stored at −70 °C until required. Cytokines were quantified using LEGENDplex™ Mouse Th17 Panel (Biolegend^®^), following the instructions provided by the manufacturer. IL-10 production was analyzed using a mouse IL-10 ELISA kit (Biolegend, San Diego, CA, USA). 

### 4.6. Immunoblotting 

Colons were flushed with PBS and frozen at –70 °C. Colon tissues were mechanically disrupted using the Bullet Blender® (Next Advance) at 4 °C and after lysis with RIPA buffer with Phosphatase Inhibitor Cocktail (PhosStop EASYpack, Roche) and Protease Inhibitor Cocktail (complete Tablets EASYpack, Roche). Tissues were centrifuged for 15 min at 4 °C and 16,000 *g*. Supernatants were collected, run on SDS gels, and transferred onto membranes. The membranes were blocked and probed with anti-Bcl-2 (Biolegend, San Diego, CA, USA) and anti-β-actin (Biolegend, San Diego, CA, USA). ImageJ was used for densitometry.

### 4.7. Flow Cytometry

Single cell suspensions from spleens and the circulation obtained during the sacrifice were washed with PBS and blocked with anti CD16/CD32 antibodies. Cells were stained with anti-CD11b, anti-Ly6C, and anti-Ly6G antibodies (Biolegend, San Diego, CA, USA) for 30 min at 4 °C. Cells were washed twice and analyzed using the FACSCalibur system and Cell Quest software (Becton Dickinson, San Jose, CA, USA).

### 4.8. Statistical Analysis

Data were analyzed either by one-way analysis of variance followed by Tukey’s multiple comparison test or by unpaired two-tailed *t*-tests with GraphPad Prism 5 (San Diego, CA, USA). All tests were performed using 95% confidence intervals. Data are expressed as means ± SEs, where * represents *p* < 0.05 and ** represents *p* < 0.01.

## 5. Conclusions

Overall, this study demonstrates that STAT1 is critical for controlling mechanisms other than apoptosis and cell proliferation during the initial stages of CAC and controls tumor growth by maintaining the balance between proinflammatory cytokines, such as IL-17 and IFN-γ, which may critically impact the recruitment of myeloid cell populations.
